# Dynamic Control of High-Range Photoresponsivity in a Graphene Nanoribbon Photodetector

**DOI:** 10.1186/s11671-020-03352-7

**Published:** 2020-06-03

**Authors:** Juan Yu, Jiahong Zhong, Xiaofei Kuang, Cheng Zeng, Lingkai Cao, Yanping Liu, Zongwen Liu

**Affiliations:** 1 School of Electronics and Information, Hangzhou Dianzi University, 1158 Second Street, Xiasha College Park, Hangzhou, 310018 Zhejiang People’s Republic of China; 2 School of Physics and Electronics, Hunan Key Laboratory for Super-microstructure and Ultrafast Process, Central South University, 932 South Lushan Road, Changsha, Hunan 410083 People’s Republic of China; 3 State Key Laboratory of High-Performance Complex Manufacturing, Central South University, 932 South Lushan Road, Changsha, Hunan 410083 People’s Republic of China; 4 Shenzhen Research Institute of Central South University, A510a, Virtual University Building, Southern District, High-tech Industrial Park, Shenzhen, 518057 People’s Republic of China; 5 School of Chemical and Biomolecular Engineering, The University of Sydney, Camperdown, Sydney, NSW 2006 Australia

**Keywords:** Graphene nanoribbons, Photodetector, Photoresponsivity

## Abstract

Graphene has been demonstrated to be a promising material for optoelectronics and photodetection devices because of its ultra-broadband optical absorption and high carrier mobility. However, its integration with optoelectronic systems has been limited by the zero-bandgap and the lack of a gain mechanism. Herein, we demonstrate a novel photodetector based on the graphene nanoribbons (GRNs) with a sizable bandgap. Utilizing trapping charge at the interface between SiO_2_ and light-doped silicon, an ultrahigh gain of 22,400 has been obtained. Our devices show an enhanced photoresponsivity (~ 800 AW^−1^) while the response speed is still fast (up to 10 μs). This photoresponsivity is about two orders of magnitude higher compared to that of a previous graphene-based photodetector. The photodetector exhibits a wide-range tunability via source-drain bias and back gate voltage. Our work addresses key challenges for the photodetectors and potentially provides the desired pathway toward practical application of graphene photodetectors that can be externally manipulated by an electric field with fast response speed and high sensitivity.

## Introduction

Graphene, a two-dimensional (2D) layered material, plays an important role in many fields including electrodialysis [1], batteries [2], nanofiltration [3], catalysis [4], electromagnetic interference [5], and optoelectronics. Significantly, graphene has attracted much attention owing to its novel optoelectronic properties [6–9], such as high carrier mobility [10, 11], zero-bandgap [12–14], and tunable Fermi level [15]. Therefore, graphene has been considered as an attractive material for optoelectronic applications [16–18]. However, the low absorption (~ 2.3%) of the monolayer graphene resulting from its thin thickness is still a critical challenge [19]. On the other hand, its zero-bandgap characteristic severely limits the optoelectronic applications, which causes a short photo-generated carrier lifetime (~ps) and results in the fast electron-hole recombination [20, 21]. As a consequence, further improvement of the responsivity of the pristine graphene photodetector remains challenging, and it is of considerable significance to separate the electrons and holes to generate an efficient photocurrent.

To overcome these challenges, various techniques have been explored and the photoresponsivity of photodetectors based on graphene has been enhanced accordingly. Photogating effect [22], which is usually observed in photodetectors based on low-dimensional materials and their hybrid structures, plays an essential role in the high performance of photodetectors. Photodetectors based on MoTe_2_ [23] and MoS_2_ [24] using the photogating effect have been reported, and photodetectors with excellent performance based on graphene utilizing photogating effect have also been achieved. It was demonstrated that combining graphene and PbS quantum dots was an effective way to enhance the absorption of light and achieve an ultrahigh gain in a graphene photodetector [25]. Besides, the recombination of electrons and holes could also be minimized in a photodetector based on heterostructures, such as graphene-Ta_2_O_5_-graphene [26], where the photoinduced electron-hole pairs were separated via quantum tunneling effects, leading to the great enhancement of the photoresponsivity and the gain. The response time of such hybrid-structure photodetector was seriously increased resulting from the long trapping time of the carriers in the PbS quantum dots or in the Ta_2_O_5_ tunnel barrier. Thus, it is highgly demanded for the graphene-based photodetector to achieve excellent performances in responsivity, response time, and spectral response.

Here, we propose a photodetector based on 20-nm-wide graphene nanoribbons and demonstrate its photoresponsivity (up to 800 AW^−1^) and fast response speed (~ 10 μs). Such high performance is mainly attributed to the sizable bandgap in the GNRs, enhanced by the photogating effect at the silicon/silicon oxide (Si/SiO_2_) interface. The physical mechanism of the detector was explained by the energy band diagrams. Furthermore, the photodetector based on GNRs can be tuned by source-drain and back-gate voltage. The observed high performance substantially paves the way for developing high-responsivity and ultrafast graphene photodetectors.

## Experimental Methods

**Fig. 1:**
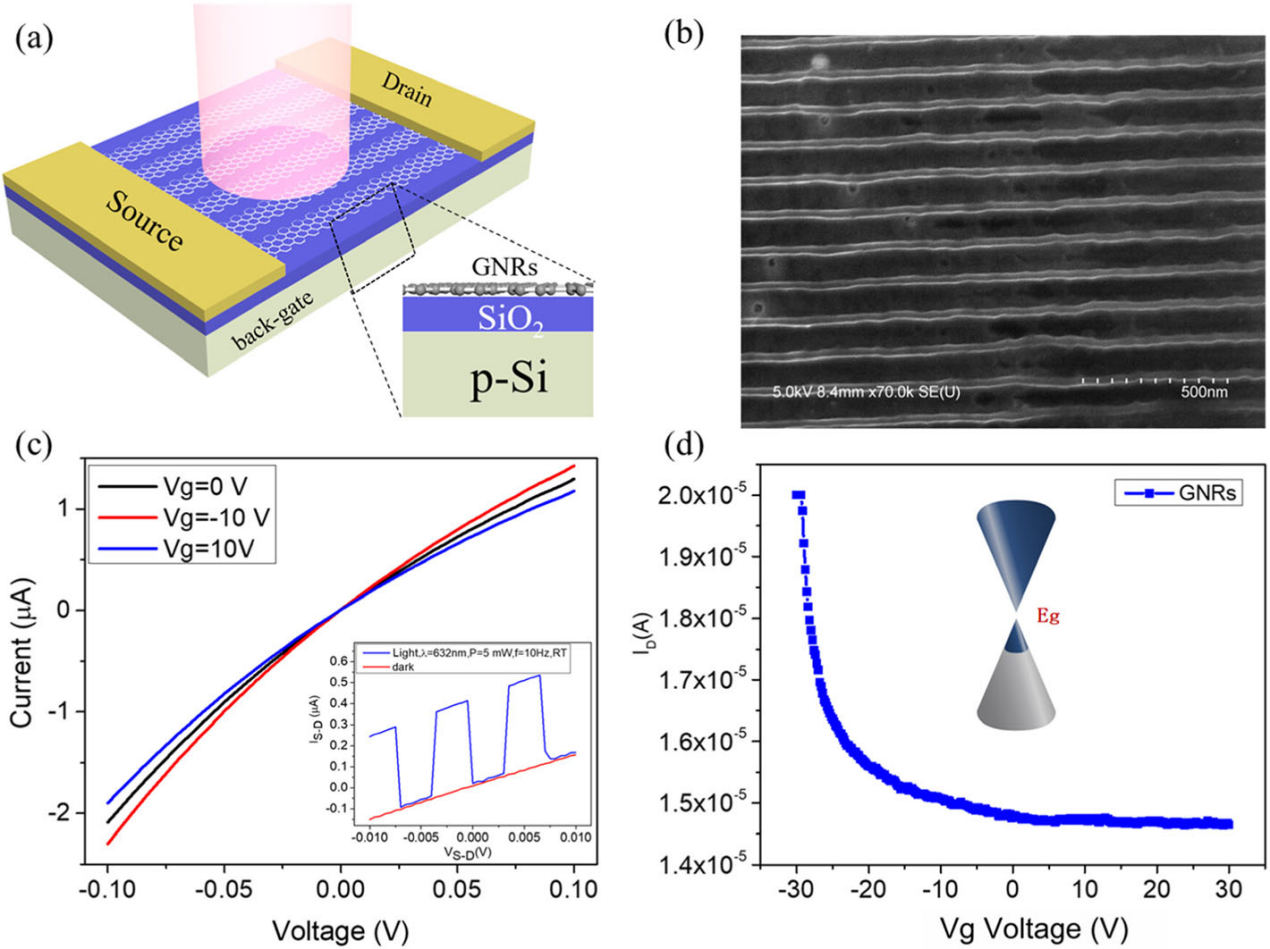
**a** A schematic illustration of the GRN photodetector. It is similar to the FET device comprised of the source and the drain electrode on the Si/SiO_2_ substrate with a lightly doped Si wafer acting as a back gate. The incident light was modulated by an optical chopper of variable frequency. **b** SEM image of the GRN photodetector device. **c** Current-voltage (I-V) characteristics of the GRN device under different back-gate voltage. Inset: I-V characteristics of the device under dark (red line) or illumination with a frequency of 10 Hz (blue line). **d** The source-drain current versus the back-gate voltage bias of GNR photodetector at room temperature. Inset: schematic diagram of GNR band structure

**Fig. 2:**
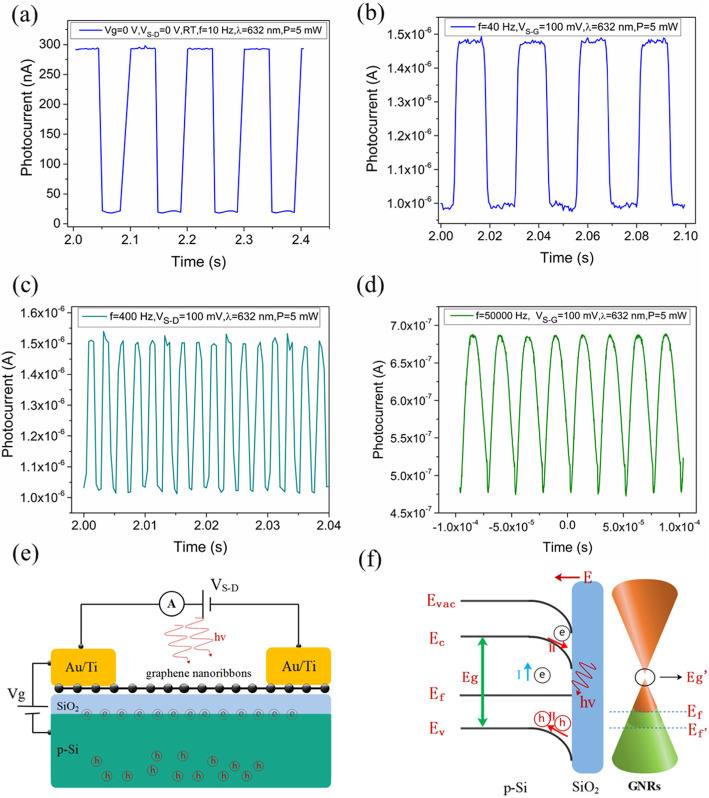
**a** Time-dependent photocurrent measurements of the device without the biased of back-gate and source-drain voltage under on-off light (632 nm) modulation at room temperature. The time-dependence photocurrent was measured under laser illumination with a frequency of 40 Hz (**b**), 400 Hz (**c**), and 5000 Hz (**d**). **e** Schematic diagram of the GNR photodetector. **f** Energy diagram of the interface between Si and SiO_2_ upon light illumination. *E*_C_, *E*_V_, *E*_fs_, and *E*_VAC_ are the conduction band, valence band, Fermi level, and vacuum level, respectively. *E*_f_ and *E*_f_’ are the Fermi level before and after the injection of the electron to the GNR channel. *E*_g_’ is the bandgap of GNRs. Two processes are illustrated: (I) electronic transition from the value band to the conduction band under illumination in Si and SiO_2_; (II) hole transfer from SiO_2_ to Si and photon excited carriers drifted through the built-in field

**Fig. 3:**
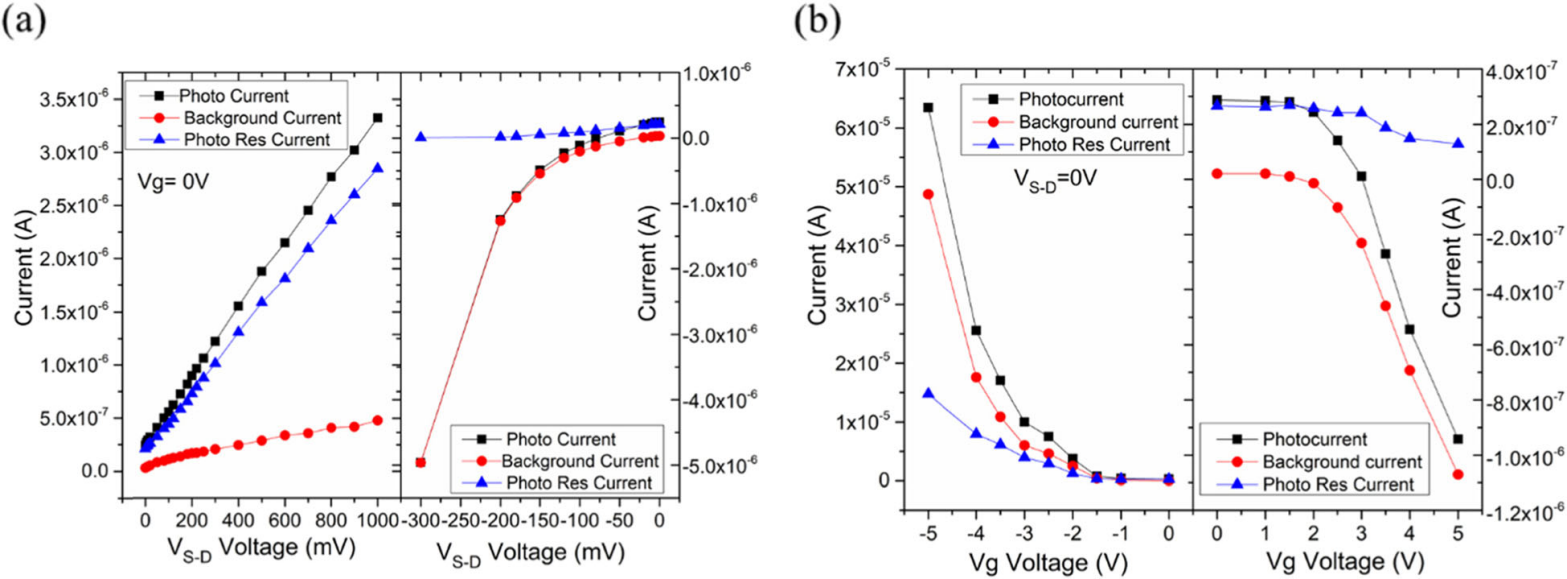
**a** Photocurrent dependence on the biased source-drain voltage. Photocurrent, background current, and photoresponse current measurements of GRN photodetector in biased of back-gate voltage. The decrease of the photocurrent with an increase of the biased source-drain voltage was contributed to the improvement of the separation efficiency of photo-generated electron-hole pairs. **b** Photocurrent dependence on the back-gate voltage. The biased back-gate voltage dependence of the photocurrent characteristics in biased of source-drain voltage. The results indicate that the photocurrent could be modulated via biasing the source-drain voltage and gate voltage

**Fig. 4:**
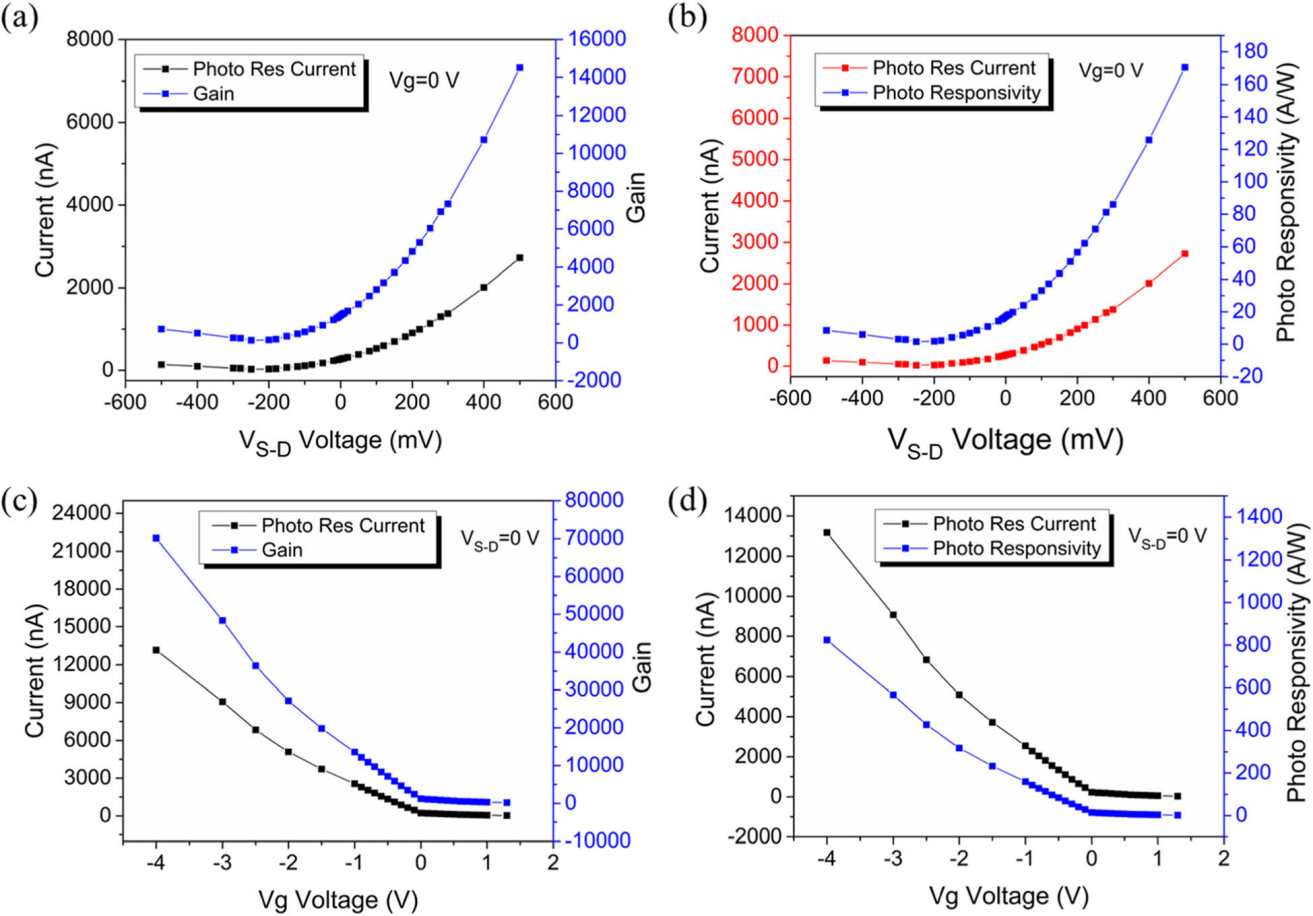
Photoresponsivity dependence on the biased source-drain voltage and gate voltage. **a** and **b** reveal the source-drain voltage dependence of photoresponsivity and gain, respectively, **c** and **d** show the back-gate voltage dependence of photoresponsivity and gain, respectively

**Fig. 5:**
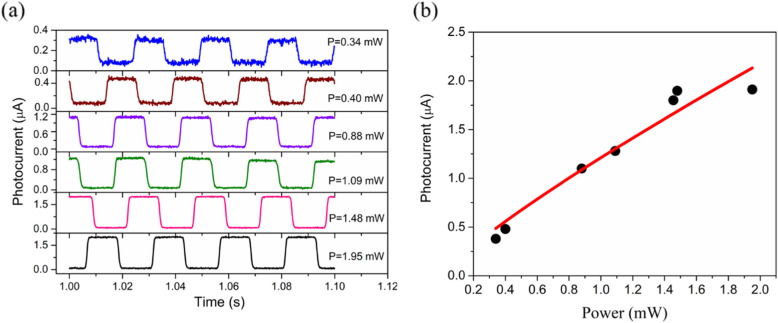
**a** The time-dependence photocurrent measurements under the different power of incident light. **b** The power dependence of the photocurrent’s properties. The results indicate that the GRN photodetector possessed a high photosensitivity property achieving mW-level input optical power detection

The graphene sheet was exfoliated onto a Si substrate (covered with 300 nm SiO_2_) from the graphite bulk (grade ZYA, SPI Supplies) by the 3M-tape micromechanical cleavage technique. Graphene nanoribbons with a width of 20 nm were fabricated using reactive-ion etching (RIE, PE-3A) and electron beam lithography (EBL, Raith BV EBPG5150). After this, the monolayer graphene and graphene nanoribbon on the SiO_2_ dielectric were characterized by an optical microscope and Raman spectroscopy (WITec Alpha 300R). Standard photolithography and e-beam evaporation of Ti/Au (20 nm/80 nm) were used to create the source and drain electrodes. Eight devices (16 GNRs) were fabricated, and 5 of them possess excellent performance. All the measurements were carried out through a home-made system composed of a laser light source, an optical chopper, a 4-probe stage, and a semiconductor parameter analyzer. A lower doped silicon (P-type 10–20 Ω cm) substrate was used to enhance the photogating effect. A Ti:Sapphire visible laser at a wavelength of around 632 nm was employed to generate laser pulses within an area of 6.25 mm^2^ at room temperature. The frequency of the incident light was modulated with an optical chopper in a range from 5 Hz to 50000 Hz. Besides, incident laser power could be adjusted from 0.34 mW to 5 mW. The data shown in the figures, including current (Figs. 1c, d, 2a–d, 3a, b, 4a–d, and 5a, b), was obtained from a semiconductor parameter analyzer (Agilent, B1500A) with or without illumination. All the photoresponse measurements were carried under ambient conditions.

## Results and Discussion

GNRs are expected to be an ideal carrier for photodetection. The GNR photodetector that we fabricated was comprised of the source and the drain electrodes on a Si/SiO_2_ substrate with a lightly doped silicon wafer acting as a back gate, as shown schematically in Fig. 1a. To ensure the high mobility and obtain a large enough bandgap simultaneously, the width of the graphene nanoribbons was chosen to be a moderate 20 nm. The complete structure of the GNRs is shown in the scanning electron microscope image (Fig. 1b), and the length of the graphene nanoribbons was 2 μm. Different from the conventional photodetectors, the lightly doped Si was adopted as the substrate for the reason that its carrier lifetime is much longer than that in the heavily doped Si [27].

The electrical characterization was repeatedly carried out and the consequent I-*V*_*S* − *D*_ relationship is plotted in Fig. 1c. The curves under different back-gate voltages in a range from – 10 V to 10 V are nonlinear and asymmetric, indicating the existence of the internal electric field, which might be resulted from the fabrication-induced defects or the Schottky barrier at the electrodes contacts. The internal electric field had a nonnegligible effect on the photocurrent of the GNR photodetector, which will be illustrated later. The inset is the comparison of I-V characteristics of the device under dark and illumination (applying a laser pulse with a frequency of 10 Hz), manifesting the sensitive optical-switching tunability. Obviously, the I-V curve shifted as the *V*_*G*_ varied. To further figure out the effect of V_*G*_ on the charge transport characteristics of the GNR channel, the transfer characteristics in the dark state were recorded at room temperature as shown in Fig. 1d. The measured *I*_*D*_ – *V*_*G*_ curve at *V*_SD_ = 10 mV demonstrated that our device displayed a typical behavior of the graphene-based photodetector, and the GNRs acted as a p-type channel with a shift of 20 V.

For typical optoelectronic systems, the response speed (characterized by the total time required for the output to rise (fall) from 10 (90)% to 90 (10)% of the pulse peak) of a photodetector determines the running speed and information capacity of the photodetection system. To investigate the ultimate response time of the fabricated device, the input optical signal with different pulse frequencies of 40 Hz, 400 Hz, and 50,000 Hz were applied. Fig. 2b–d show the corresponding time-resolved total photocurrents, which intuitively reflect that the fabricated photodetector could be efficiently switched on and off with excellent repeatability. Furthermore, when the laser frequency was adjusted to 50,000 Hz, the rising time was measured to be 10 μs. We believe that our device is expected to operate at higher frequencies over 50,000 Hz, and the accurate value of response speed is not clear due to the limitation of the measuring equipment. It was noted that the GNR photodetector run much faster than most photodetectors based on graphene and other 2D TMDs [28–31]. It is believed that the fast photocurrent switching can be attributed to the ultrahigh carrier mobility of the GNRs of such width and the strong external electric field.

In addition to the fast response speed, high responsivity and enhanced gain are indispensable for the application of the photodetector. Therefore, through applying light on the entire device at room temperature, we further studied the photoresponse of the GNR photodetector without source-drain bias and back-gate voltage. Figure 2a presents the time-dependent photocurrent measurements of the device in the absence of the biased voltage under on-off light modulation. The observed photocurrent was 275 nA(*I*_illumination_ = 293 nA, *I*_dark_ = 18 nA) under illumination, which indicated a high photoresponsivity of *R* = 17.2 AW^−1^ and a high gain of *G* = 1465 as well, calculated via the following two equations:

1 $$ R=\frac{I_P}{\frac{S_G}{S_L}\cdotp P} $$

2 $$ G=\frac{I_P/e}{\left(\frac{S_G}{S_L}\cdotp P\cdotp 2.3\%\right)/ h\nu}\left(\nu =\frac{c}{\lambda_{in}}\right) $$

where *I*_*P*_ (275 nA) is the photocurrent, while *S*_*L*_ (6.25 mm^2^) and *S*_*G*_ (2 μm×10 μm) are the actual area of the laser and the GNR, respectively, and *P* (5 mW) is the power of incident laser with a wavelength of *λ*_*in*_ (532 nm). It is essential to explore the photocurrent generation mechanism of the GNR photodetectors to clarify the high performance of our devices. For photodetectors based on two-dimensional materials, there are mainly two photocurrent generation mechanisms: the photoconductive effect (PC) and the photovoltaic effect (PV) [32].

Without applying a source-drain bias, PV was responsible for the photocurrent generation as the two built-in electrical fields were formed between the GNRs and the electrodes. The two electrical fields were not the same magnitude due to defects formed in the manufacturing process. When the light reached the region at the Au-GNRs interface, the photo-generated electron-hole pairs were generated and subsequently separated by the built-in fields, which made a significant contribution to the photocurrent generation. Under a source-drain bias, however, the two built-in electrical fields at the Au-GNRs interface played little part in photocurrent generation. Therefore, PC played the most crucial role in the photocurrent generation in the case of applying a source-drain bias. After absorbing photons, the GNR channel generated more free carriers, reducing the resistance of the carrier channels. Therefore, a significant photocurrent *I*_*P*_= $\frac{V_{OC}}{R_G}$( *V*_*OC*_ represents the open-circuit voltage and *R*_*G*_ is the total resistance of the channel formed by the 16 graphene nanoribbons) was observed.

As can be seen in Fig. 2a–d, a *μA*-level photocurrent was observed, which might be due to the contribution of three aspects. One was that the electron-hole pair recombination rate was reduced resulting from the bandgap in the GNRs. The other was that the photogenerated electrons were captured during the transition from the valence band to the conduction band by the midgap states [33] induced by the edge defects of GNRs. Therefore, before the holes and the trapped electrons recombined, the holes could circulate between the drain-source electrodes to form the photocurrent, achieving a high gain. The third aspect was that the accumulation of electrons at the SiO_2_/Si interface was equivalent to applying a vertical electric field, and thus the conductance of the channel was greatly enhanced. Furthermore, in Fig. 2a–d, the obtained photocurrent had little dependence on the frequency of incident light modulated by an optical chopper, which is similar to the reported MoS_2_ photodetector [24]. The photoconductive effect played the primary role in the photocurrent generation of the GNR photodetector when the frequency of light was regulated by the chopper. However, when the device was exposed to light (0 Hz), the photogating effect would be significant in the process of carriers generation, leading to trapping and recombination within semiconductors.

The detailed physical process of the third aspect discussed above was demonstrated in Fig. 2e, f. To achieve an equilibrium state in the dark, electrons would diffuse from SiO_2_ to Si due to the difference of Fermi levels between the two materials, which led to energy band bending at the Si/SiO_2_ interface. As a result, a strong built-in electric field (E) was formed in the depletion region, which efficiently separated the photogenerated electron-hole pairs with the electrons moving to the interface between Si and SiO_2_ while the holes transfering to the interior region of Si. The electrons then accumulated at the SiO_2_/Si interface, and these trapped electrons applied an additional negative vertical voltage to the GNRs, where the presence of these electrons increased the hole concentration and lowered the Fermi level of the GNR channel accordingly.

Although the device displays high performance, it is of importance to seek several effective approaches to significantly boost the photocurrent and the responsivity of the device. Then, the effects of the source-drain bias and gate voltage on photocurrent were systematically investigated. Figure 3a shows results of photocurrent (*I*_laser_), background current (*I*_dark_), and photoresponse current (*I*_ph_) measurements as a function of the source-drain voltage (− 3 V ≤*V*_*S* − *D*_ ≤ 10 V) at a fixed gate voltage. The photocurrent was not zero at *V*_*S* − *D*_ = 0 and increased nonlinearly with the source-drain voltage, also proving the existence of a built-in electric field. It is clear that the value of the photocurrent was strongly dependent on the source-drain bias.

A convincing explanation for the tunability via source-drain voltage is that the relationship between photocurrent, background current, and photoresponse current can be expressed as *I*_illumination_ = *I*_ph_ + *I*_dark_, where *I*_ph_ and *I*_dark_ increased with drain-source voltage *V*_*S* − *D*_ because the drift velocity of carriers rised and the carrier transit time reduced under an external electric field [34]. Therefore, the separation efficiency of photogenerated carriers improved, significantly contributing to the large photocurrent. Such phenomenon indicates that the total electric field of the GNR channel, the sum of the internal electric field and the external electric field, can be modulated by *V*_*S* − *D*_.

Moreover, considering the gate-tuneable carrier density of GNR, the photocurrent of our device was adjusted effectively by modulating the back-gate voltage. Figure 3b displays these three kinds of currents (*I*_illumination_, *I*_ph_ and *I*_dark_ ) as a function of the back-gate voltage (− 5 V ≤ V_*G*_ ≤ 5 V) at *V*_*S* − *D*_ = 0. In general, the photocurrent was positively correlated with the absolute value of the gate voltage, because the carrier density of GNR was sensitive to the external vertical electric field. Interestingly, the photocurrent increased as the gate voltage increased when the gate voltage was negative (− 5 V ≤V_*G*_ ≤ 0 V), and the opposite occured when the gate voltage was positive (0 V ≤V_*G*_ ≤ 5 V). This phenomenon could be explained by the p-type behavior of the GNR channel, which agreeed well with the observation in Fig. 2d. The results indicate that the increased |V_*G*_| can tune the Fermi level of the channel closer to the valence band (or conduction band) and the conductance of the GNR channel was gate-tuneable. Notably, for both the two modulation methods (source-drain voltage and back-gate voltage), the tunability of the photocurrent was demonstrated in an ultrawide range from nA-level to μA-level.

Additionally, the responsivity and gain could also be modulated efficiently by regulating the gate voltage and the source-drain voltage of the GNR photodetector. The gain and photoresponsivity dependence of the source-drain bias were calculated [according to Eqs. (1) and (2)] and subsequently plotted in Fig. 4a, b. For the photodetector based on GNRs, the relationship between the gain and *V*_*S* − *D*_ is given by the following formula:

3 $$ G=\frac{\tau }{\tau_T}=\frac{\tau }{l^2/\left(\mu {V}_{S-D}\right)}=\frac{\tau \mu {V}_{S-D}}{l^2} $$

where *τ* is excess hole lifetime (trapped hole lifetime), and *τ*_*T*_ = *l*^2^/(*μV*_*S* − *D*_) is the transit time of the carrier, while *l* is the length of the channel and *μ* is the carrier mobility, whereas *V*_*S* − *D*_ is the source-drain bias. Hence, the gain and source-drain voltage exhibit a positive correlation. Apparently, *G* is linearly dependent on the source-drain bias. As a result, the maximum photoresponsivity of *R* = 170 AW^−1^ and the maximum gain of *G* = 14,500 were achieved at room temperature at *V*_*S* − *D*_ = 0.5 V, which was a 100-fold improvement over previous graphene-nanostructure-based photodetectors [26, 35, 36]. More importantly, the values of gain and photoresponsivity were not saturated. Consequently, a higher gain and photoresponsivity could be achieved if a larger drain-source voltage was applied.

Figure 4c, d shows that the photoresponsivity and gain could also be enhanced by applying a back-gate bias to improve the carrier concentration of the GNRs. The maximum photoresponsivity of *R* = 800 AW^−1^ and the maximum gain of *G* = 22400 were obtained at V_*G*_ = - 4 V. This maximum value of photoresponsivity was five orders of magnitude higher than that of pure graphene photodetectors (~ 10 mAW^−1^) [37]. Furthermore, both gain and photoresponsivity were not saturated, therefore, a higher photoresponsivity could be achieved by applying a larger back-gate voltage. Besides the carrier concentration, another factor that significantly influenced the channel current was the contact resistance (*R*_*C*_) between the Au electrodes and the GNRs which was inseparably related to the Schottky barrier height at the interface [34]. As the GNRs served as a p-type channel, when applying a negative *V*_G_, the Schottky barrier height was reduced due to the lower Fermi level. In contrast, when the *V*_*G*_ was increased to a positive value, the Schottky barrier height was enhanced, and the current in the channel was greatly suppressed.

Finally, we turn to the time-dependence investigation of photocurrent under incident light of power. Figure 5a displays time-dependent photocurrent measurements under the different powers of incident light. This photocurrent was large enough for direct measurement without any current preamplifiers or lock-in amplifiers, even at an mW-level optical power. Figure 5b plots the photocurrent as a function of incident optical power. The photocurrent had a nonlinear relationship with incident power (*I*_ph_ = P^α^, α = 0.85). Under lower light power, the contribution of the photogate current was dominant, and the photoconductive effect could be ignored due to a decrease in the number of photogenerated carriers [23]. Upon higher light illumination, on the contrary, an increasing current was observed, which could be attributed to the increased number of photogenerated electrons (photoconductive effect). Moreover, the device was sensitive to the incident light and the resulted photocurrent was closely related to the incident light energy, revealing the tremendous potential for optical power monitor. A comparison of optoelectronic parameters in various photodetectors is provided in Table 1.

**Table 1:** Comparison of critical performance indicators in photodetector based on 2D materials

Materials	Responsivity(A/W)	Response time(μs)	λ(nm)	Ref.
Graphene nanoribbons	800	10	532	This work
Graphene	32	150	532	[38]
Graphene nanoribbons	1.75	> 2 × 10^6^	1470	[39]
1 L MoS_2_	7.5 × 10^−3^	5 × 10^4^	400–1000	[40]
1 L MoSe_2_	97.1	1.5 × 10^5^	532	[28]
1 L WS_2_	9.2 × 10^−5^	5.3 × 10^3^	Visible	[41]

## Conclusions

In summary, we have demonstrated a high-performance graphene nanoribbon photodetector modulated in a wide range through the external electrical field at room temperature. Meanwhile, without the external electric field, the performance of the device could be enhanced by the localized field at the Si/SiO_2_ interface. The device exhibited a high photoresponsivity of 800 AW^−1^ at *V*_*G*_ = − 4 V, which was two orders of magnitude higher than those in the previous research. Furthermore, the structure of our device is much simpler compared to the previous graphene-based optoelectronic device with the potential broad applications. The performance of the graphene nanoribbon device can be further improved by h-BN encapsulation, surface plasmons, ferroelectric field, and hybrid structures. The proposed graphene nanoribbons photodetector opens up exciting opportunities for ultrafast and high sensitivity for future graphene-based safety monitoring, photo-communication, and aviation applications.
